# Deep Learning Based Prediction on Greenhouse Crop Yield Combined TCN and RNN

**DOI:** 10.3390/s21134537

**Published:** 2021-07-01

**Authors:** Liyun Gong, Miao Yu, Shouyong Jiang, Vassilis Cutsuridis, Simon Pearson

**Affiliations:** 1 School of Computer Science, University of Lincoln, Lincoln LN6 7TS, UK; myu@lincoln.ac.uk (M.Y.); sjiang@lincoln.ac.uk (S.J.); vcutsuridis@lincoln.ac.uk (V.C.); 2 Lincoln Institute for Agri-Food Technology, University of Lincoln, Lincoln LN6 7TS, UK; SPearson@lincoln.ac.uk

**Keywords:** deep learning, temporal convolutional network (TCN), recurrent neural network (RNN), crop yield prediction, greenhouse

## Abstract

Currently, greenhouses are widely applied for plant growth, and environmental parameters can also be controlled in the modern greenhouse to guarantee the maximum crop yield. In order to optimally control greenhouses’ environmental parameters, one indispensable requirement is to accurately predict crop yields based on given environmental parameter settings. In addition, crop yield forecasting in greenhouses plays an important role in greenhouse farming planning and management, which allows cultivators and farmers to utilize the yield prediction results to make knowledgeable management and financial decisions. It is thus important to accurately predict the crop yield in a greenhouse considering the benefits that can be brought by accurate greenhouse crop yield prediction. In this work, we have developed a new greenhouse crop yield prediction technique, by combining two state-of-the-arts networks for temporal sequence processing—temporal convolutional network (TCN) and recurrent neural network (RNN). Comprehensive evaluations of the proposed algorithm have been made on multiple datasets obtained from multiple real greenhouse sites for tomato growing. Based on a statistical analysis of the root mean square errors (RMSEs) between the predicted and actual crop yields, it is shown that the proposed approach achieves more accurate yield prediction performance than both traditional machine learning methods and other classical deep neural networks. Moreover, the experimental study also shows that the historical yield information is the most important factor for accurately predicting future crop yields.

## 1. Introduction

Compared with field growing, currently, greenhouse growing is preferred by many crop growers. Growing crops in the greenhouse can extend their growing season, protect crops against temperature and weather changes and thus provide a safe growing environment. Moreover, environmental parameters (e.g., humidity, temperature radiation, carbon dioxide, etc. [1,2] can also be controlled in the modern greenhouse to guarantee crops grow at the most appropriate environmental conditions.

Crop yield forecasting in greenhouses plays an important role in farming planning and management in greenhouses, and optimally controlling environmental parameters guarantees the maximum crop yield. Cultivators and farmers can utilize yield prediction in greenhouses to make knowledgeable management and financial decisions. However, it is an extremely challenging task. There are many factors that have an influence on crop yield in a greenhouse, such as radiations, carbon dioxide concentrations, temperature, quality of crop seeds, soil quality and fertilization, and disease occurrences (as shown in [3,4,5]). It is not straightforward to construct an explicit model to reflect the relationship between such a variety of factors and crop yield.

In this work, we propose a deep neural network-based greenhouse crop yield prediction method, by combining two state-of-the-art networks for temporal sequence processing: recurrent neural network (RNN) and temporal convolutional network (TCN). The proposed deep neural network is developed for predicting future crop yields in a greenhouse based on a sequence of historical greenhouse input parameters (e.g., temperature, humidity, carbon dioxide, radiation) as well as yield information. According to the experimental evaluations of multiple datasets collected from multiple greenhouses in different time periods, it is shown that the RNN+TCN-based deep learning approach achieves more accurate yield prediction results with smaller root mean square errors (RMSEs), compared with both classical machine learning and other popular deep learning-based counterparts.

## 2. Literature Works

Although there is much research related to crop yield prediction for the farming field, a relatively small amount of works focus on greenhouse crop yield forecasting. Approaches that have been developed for greenhouse crop yield forecasting are divided into two main categories: the explanatory biophysical model-based approach and the data driven/machine learning model-based approach.

Explanatory biophysical model-based approach: Based on a series of ordinary differential equations (ODEs) reflecting a dynamic process, the explanatory model describes the relationship between some environmental factors and crop growth or morphological development. Different biophysical models have been applied for crop growth modelling which can thus be used for yield forecasting, based on greenhouse environmental parameters.

The Tomgro model is proposed by Jones et al. in [6], which models the tomato growth and fruit yield with respect to dynamically changing temperature, solar radiation, and CO${}_{2}$ concentration inside a greenhouse. A more complex Tomsim biophysical model is proposed in [7], which contains multiple sub-modules developed for modelling different aspects (i.e., photosynthesis, dry matter production, truss appearance rate, fruit growth period and dry matter partitioning, etc.) related to tomato growth. A crop yield model that describes the effects of greenhouse climate on yield based on ODEs was described and validated in [4]. This yield model was validated for four temperature regimes. Results demonstrated that the tomato yield was simulated accurately for both near-optimal and non-optimal temperature conditions in the Netherlands and southern Spain, respectively, with varying light and CO${}_{2}$ concentrations. An integrated Yield Prediction Model [8], which is an integration of Tomgro model [6] and Vanthoor model [4], is applied to predict the crop yield in greenhouses based on controllable greenhouse environmental parameters. Different biophysical models, including Vanthoor model [4], Tomsim model [7], Greenhouse Technology applications (GTa) model, the model proposed in [9] and their combined version were compared in [10]. The experimental studies show that the combined model can outperform original models with smaller root mean square errors (RMSEs) for yield prediction. The biophysical models proposed in [11,12] describe effects of electrical conductivity, nitrogen, phosphorus, potassium, and light quality on dry matter yield and photosynthesis of greenhouse tomatoes and cucumbers, respectively.

The explanatory model is practical to reflect the actual growth process of crops, which is bio-physically meaningful and explainable. However, the aforementioned explanatory models suffer from the following two main limitations:

(i) There are many intrinsic model parameters associated with a biophysical model and the performance of an explanatory model is highly sensitive to its model parameters (as shown in [13]). Moreover, the model parameter setting suitable for predicting greenhouse crop yield in one region may not be workable for other regions [13].
(ii) In addition, most biophysical models are also not universal and restricted to model the growth for a specific type of plant. For example, the Tomgro model [6] and Tomsim model [7] can only be used to model/predict the growth/yield of tomatoes.

Due to the limitations of the explanatory biophysical model-based approaches, in this work, we refer to another category of approach–machine learning model-based approach for greenhouse crop yield prediction. More details of the machine learning model-based approach are introduced as follows.

Machine learning model-based approach: Data driven/machine learning technique-based approaches have also been applied for greenhouse crop yield forecasting in many studies, which treat the crop yield output as a very complex and nonlinear function of the greenhouse environmental variables and historical crop yield information. In particular, linear and polynomial regression models are used in [14] for strawberry growth and fruit yield using environmental data such as average daily air temperature (ADAT), relative humidity (RH), soil moisture content (SMC), and so on. However, an assumption of a linear or polynomial relationship between the crop yield and environmental factors is not always valid. Partial least squares regression (PLSR) has been applied in [15], for modelling the yield of snap bean based on the data collected from hyperspectral sensing. Neural networks have also been widely applied for greenhouse crop yield prediction. For example, an artificial neural network (ANN) has been applied in [16], for weekly crop yield prediction. While in [17], ANN has been applied to predict the pepper fruit yield based on factors such as fruit water content, days to flowering initiation, and so on. An Evolving Fuzzy Neural Network (EFuNN) was proposed in [18] for automatic tomato yield prediction, given different environmental variables inside the greenhouse, namely, temperature, CO${}_{2}$, vapour pressure deficit (VPD), and radiation, as well as past yield. A Dynamic Artificial Neural Network (DANN) [19] was implemented to predict tomato yields, based on a series of predictors such as CO${}_{2}$ fixation, transpiration, solar radiation as well as past yield. The findings show that the most important environmental variable for yield prediction was CO${}_{2}$ fixation, and the least important was transpiration. Although ANN-based approaches have been widely applied for greenhouse crop yield prediction tasks as in [16,17,18,19], their performance is highly sensitive to different choices of network architectures and network hyper-parameters settings. Furthermore, there is a lack of studies on optimally designing network architecture and tuning network hyper-parameters for the greenhouse crop yield prediction.

The aforementioned works focus on using classical machine learning approaches for greenhouse crop yield prediction. Given a certain amount of training data, classical machine learning models (such as linear/polynomial regression models, artificial neural network model, etc.) are constructed to predict greenhouse crop yields based on certain factors (such as environmental and past yield information). However, these works suffer from limitations due to the adoptions of simple and ‘shallow’ classical machine learning models, for example:

(i) Features extracted from data for building the classical machine learning models may not be optimal and most representative, thus deteriorating the performance for yield prediction (as shown by our experiment, in most cases, the classical machine learning models perform worse than the deep learning-based ones).
(ii) The classical machine learning models cannot effectively handle data with either high volume or high complexity.

Deep learning is a very popular machine learning technique and it has been successfully applied in a variety of applications (e.g., image classification, computer vision, natural language processing, etc.) [20]. Recently, deep learning technology has also been applied for crop yield prediction in the outdoor environment. For example, in [21], a recurrent neural network deep learning algorithm over the Q-Learning reinforcement learning algorithm is used to predict the crop yield. The results show that the proposed model outperforms the existing models with high accuracy for crop yield prediction. CNN and LSTM are combined in [22] for both end-of-season and in-season county-level soybean yield prediction, based on the remote sensing data in the outdoor environment. Compared with the outdoor application scenarios, there are very few works related to the applications of the deep learning approach for indoor greenhouse crop yield prediction. Some related works can be found in [5,23], from which the researchers have adopted the recurrent neural network (RNN) model with long-short temporal memory (LSTM) units for tomato and ficus yield prediction. Furthermore, it can be seen from the evaluation results that the deep learning-based approaches adopted in [5,23] outperform traditional machine learning algorithms, with more accurate prediction results and lower root mean square errors (RMSEs).

## 3. Methodology

**Figure 1:**
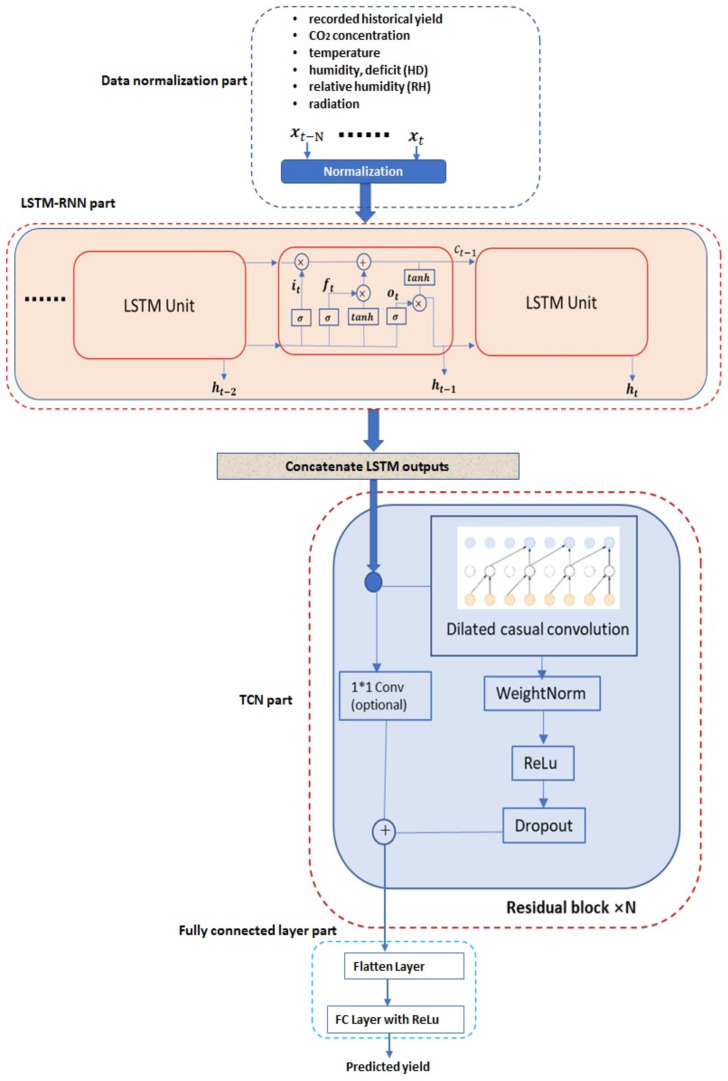
Proposed DNN architecture.

In this work, a novel deep neural network (DNN)-based methodology is proposed, to predict the future crop yield based on historical yields and greenhouse environmental parameters (e.g., CO${}_{2}$ concentration, temperature, humidity, radiation, etc.) information. The proposed method is based on the hierarchical integration of the recurrent neural network (RNN) and temporal convolutional network (TCN), which are both the current state-of-the-art DNN architectures for temporal sequence processing. Furthermore, a diagram illustrating the proposed methodology is shown in Figure 1, from which we can see that the proposed methodology contains four main parts: normalization part, recurrent neural network part, temporal convolutional network part and the final fully connected layer part. Different parts will be introduced in the next few sections.

### 3.1. Input Data Normalization

A temporal sequence of data containing both historical yield and environmental information is exploited to predict the future crop yield after a certain period. As shown in Figure 1, a temporal sequence with the length *N* denoted as $x_{t-N},\;$…$,\;x_{t}$ is taken as the network input. The $x_{t}$ in the temporal sequence is a vector containing the following factors recorded at the time instance *t*: recorded yield information (g/m${}^{2}$), CO${}_{2}$ concentration (ppm) in the greenhouse, greenhouse temperature (${}^{\circ }$C), humidity deficit (g/kg), relative humidity (percentage) and radiation (W/m${}^{w}$).

Before being fed into the network, firstly, normalization is applied to the data to normalize each factor (e.g., historical yield, CO${}_{2}$ concentration, temperature, radiation, etc.) to a range between [0, 1] by the following equation:

(1) $$\begin{array}{c} {\hat{x}}_{t}^{i}=\frac{x_{t}^{i}-x_{min}^{i}}{x_{max}^{i}-x_{min}^{i}} \end{array}$$

where $x_{t}^{i}$ represents the *i*-th factor at the time step *t*. $x_{min}^{i}$ and $x_{max}^{i}$ represent the corresponding maximum and minimum values for the related factor. After applying Equation (1), each factor is normalized to a range between 0 and 1.

### 3.2. Recurrent Neural Network

The normalized temporal data sequence is then fed into a recurrent neural network. As in [20], RNN has been widely applied for processing sequence data. It can both capture temporal dependencies between data samples in a sequence and extract the most representative features for that sequence to perform a variety of tasks (e.g., sequence classification, temporal data predictions, etc.). In our work, the RNN is firstly applied to extract representative features from input normalized temporal sequence data for further processing.

The traditional RNN exists problems of both gradient vanishing and gradient explosion [20], which limits its applications especially on processing long sequential data. Currently, the most popular way to overcome limitations of the traditional RNN is to adopt a new architecture with incorporating long short-term memory (LSTM) units, known as LSTM–RNN [24]. As shown in Figure 1, the LSTM–RNN consists of multiple LSTM units, which are shown in Figure 1. Furthermore, there are a series of arithmetic operations associated with a LSTM unit, which are detailed as follows:

(2) $$\begin{array}{cc} & i_{t}=\sigma \left(W_{xi}x_{t}+W_{hi}h_{t-1}+W_{ci}c_{t-1}+b_{i}\right)\\ & f_{t}=\sigma \left(W_{xf}x_{t}+W_{hf}h_{t-1}+W_{cf}c_{t-1}+b_{f}\right)\\ & c_{t}=f_{t}c_{t-1}+i_{t}tanh\left(W_{xc}x_{t}+W_{hc}h_{t-1}+b_{c}\right)\\ & o_{t}=\sigma \left(W_{xo}x_{t}+W_{ho}h_{t-1}+W_{co}c_{t-1}+b_{o}\right)\\ & h_{t}=o_{t}tanh\left(c_{t}\right) \end{array}$$

where $x_{t}$, $o_{t}$ and $h_{t}$ represent the LSTM input, LSTM output and LSTM state associated with the data sample at time instance *t*. $c_{t}$ is the LSTM cell value representing encoded historical information obtained from previous data samples before *t*. σ(·) and tanh(·) represent sigmoid and tanh functions. Other parameters represent weights and bias.

Given the normalized input temporal sequence as shown in Figure 1, representative features are extracted by the LSTM–RNN network as its states $\left[...,h_{t-1},h_{t},h_{t+1},...\right]$, which are then fed into the next component of temporal convolutional network (TCN) for further processing.

### 3.3. Temporal Convolutional Network

The temporal convolutional network (TCN) component used in this work, as proposed in [25], applies a hierarchy of temporal convolutions across its input sequence, thus effectively extracting its representative features from different temporal scales. As in Figure 1, the dilated TCN component consists of multiple residual blocks while each residual block consists of multiple dilated causal convolution layers. Dilated causal temporal convolution operations are performed in the dilated convolution layers. In particular, the *t*-th output in the *l*-th layer and *j*-th block (denoted as $S_{t}^{j,l}$) is calculated from the previous layer by the following operations:

(3) $$\begin{array}{c} S_{t}^{j,l}=f\left(w_{1}S_{t-s}^{j,l-1}+w_{2}S_{t}^{j,l-1}+b\right) \end{array}$$

where f(·) represents the activation function (such as Relu as shown in Figure 1), $w_{1}$ and $w_{2}$ represent weights and b is the bias value. During the training procedure of the dilated convolution layers, weight normalization [26] can be performed on the dilated convolution layer’s weights, to help speed up the convergence of the related weights training algorithms. Moreover, a certain percentage of weights of the dilated convolution layers can be dropped out during the training, for improving the generalization performance.

An additional 1D convolution operation is performed within each residual block, to adjust the dimension of the residual block input to be the same as that of the dilated casual convolution layer output to add them together. The output results obtained from one residual block are fed as the input of the next block and the final output is obtained from the last residual block. The final output of the last residual block from the TCN is then flattened and fed into a fully connected (FC) layer to output the final yield prediction result.

### 3.4. Fully Connected Layer

The output of the TCN part is flattened and fed into a vector, which is then fed into a fully connected layer for the final yield prediction, as shown in Figure 1. In particular, the fully connected layer has one output with a Relu activation function.

In a summary, the proposed work investigates the combination of two state-of-the-arts deep neural networks for temporal sequence processing: LSTM–RNN and TCN, for greenhouse crop yield prediction. Based on an input temporal sequence containing both historical yields and environmental parameters information during a certain period, firstly, an LSTM–RNN is applied for pre-processing the original input to extract representative feature sequences, which are then further processed by a sequential of residual blocks in the TCN to generate the final features used for the future yield prediction. Compared with other deep learning methodology for greenhouse crop yield prediction by solely exploiting the LSTM–RNN as in [5], in our work, an additional TCN layer is added on the top of the LSTM–RNN layer, to better exploit the LSTM–RNN output and extract more representative features for a more accurate crop yield prediction. As validated from the experimental studies, the combination of RNN and TCN achieves better performance than exploiting solely RNN [5] or TCN for the greenhouse crop yield prediction.

## 4. Experimental Studies

The experimental studies of the proposed DNN based crop yield prediction approach are presented in this section.

### 4.1. Datasets Descriptions

**Figure 2:**
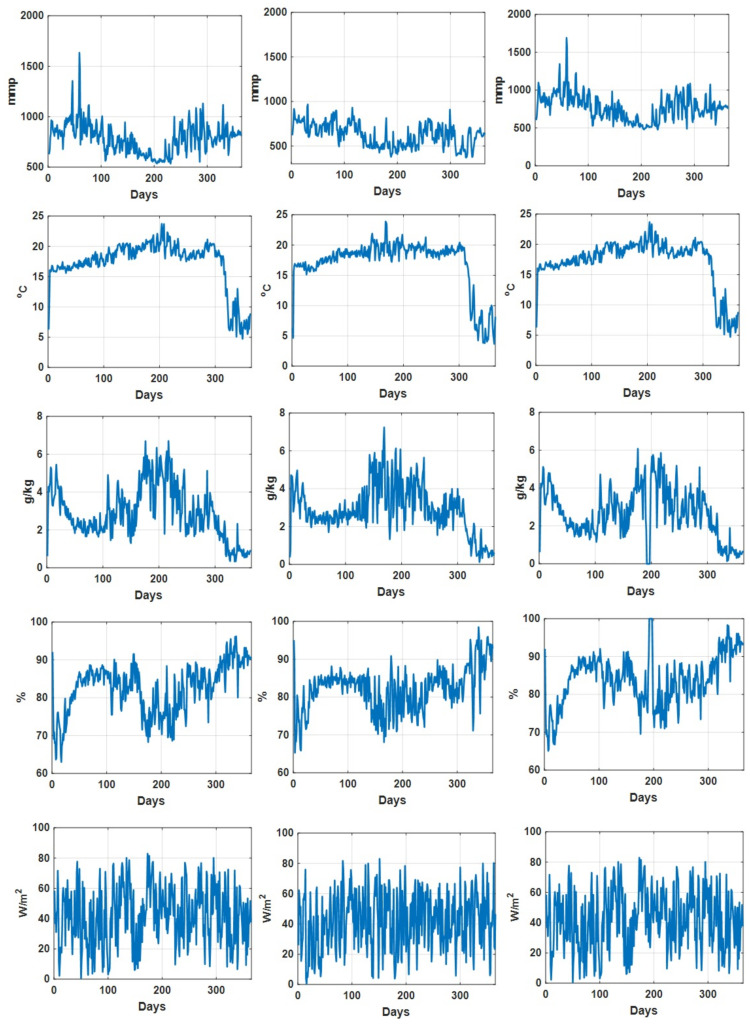
Daily recorded CO${}_{2}$ concentration (mmp), temperature (${}^{\circ }$C), humidity deficit (g/kg), relative humidity (percentage) and radiation (W/m${}^{2}$) associated with dataset 1 (**left column**), dataset 2 (**middle column**) and dataset 3 (**right column**).

**Figure 3:**
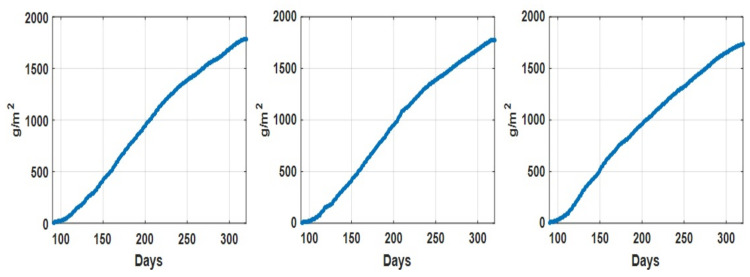
Accumulated tomato fruit yield (g/m${}^{2}$) recorded associate with dataset 1 (**left**), dataset 2 (**middle**) and dataset 3 (**right**).

Three datasets are collected from a tomato-growing site in Newcastle, UK, which contain recorded environmental parameters and crop yield information in different greenhouses during different time periods. The details of these datasets are described in the following Table 1:

As an illustration, the daily recorded environmental parameters (CO${}_{2}$ concentration, temperature, humidity deficit, relative humidity, and radiation) for all three datasets are shown in Figure 2. In addition, the descriptive statistical analysis on environmental parameters for different datasets is summarized in Table 2. We can see that for Dataset 2, the descriptive statistics (min, max median, and mean values) of recorded CO${}_{2}$ concentration values are comparatively lower than those in the other two datasets, while the descriptive statistics of the other environmental parameters for these three datasets are quite consistent. Figure 3 shows the recorded accumulated tomato yield information during a one year period associated with three datasets. Furthermore, we can see that the recorded accumulated dry fruit weights follow similar patterns (due to the fact that they are from one grower at one particular site).

### 4.2. Experimental Design

Based on the temporal recordings of environment and yield information for every dataset, the sliding window method (with step 1) is applied to generate data samples, which contain recorded environmental parameters and yield information during one week as well as the associated future crop yield after one week. These generated data samples are exploited to train/test a network, for predicting the crop yield after one week given the collected environmental and yield information during the last week. Generated data samples are split into training and testing datasets with a proportion of 70% versus 30%. The training dataset is applied to train the network, which is then tested against another testing dataset for evaluating the performance of the trained network. Adam’s method in [27] is applied for network training to minimize the mean square error (MSE) loss defined as below:

(4) $$\begin{array}{c} L_{MSE}=\frac{1}{N_{train}}\Sigma_{i=1}^{N}{∥y_{i}^{train}-{\hat{y}}_{i}^{train}∥}^{2} \end{array}$$

where $N_{train}$ is the train sample size while $y_{i}^{train}$ and ${\hat{y}}_{i}^{train}$ represent the ground truth and predicted yield values in the training dataset, respectively.

Furthermore, we use the root mean square error (RMSE) as defined below to evaluate the network performance on a testing dataset

(5) $$\begin{array}{c} RMSE=\sqrt{\frac{1}{N}\Sigma_{i=1}^{N}{∥y_{i}^{test}-{\hat{y}}_{i}^{test}∥}^{2}} \end{array}$$

where $N_{test}$ is the sample size in the test dataset while $y_{i}^{test}$ and ${\hat{y}}_{i}^{test}$ represent the ground truth and predicted test sample values, respectively.

### 4.3. Network Performance

For evaluating the proposed deep neural network’s performance for the greenhouse crop yield prediction, firstly, we need to identify the optimal network architecture. As mentioned in the previous section, our developed DNN is combined with two components: LSTM–RNN and TCN. The LSTM–RNN component consists of multiple LSTM units while the TCN component contains residual blocks containing three dilated convolutional layers. Each convolutional layer contains multiple convolutional filters with kernel size 2 and dilated rate 1 in [25]. Firstly, we have evaluated different network architectures with different LSTM units and convolutional filter numbers. Especially, each network architecture is trained/tested based on the training/testing split of data samples associated with every dataset multiple times, while the mean and standard deviation of obtained multiple RMSEs are calculated. The calculated means and standard deviations of RMSEs associated with different network architectures for all three datasets, as well as average ones (calculated as the average of the RMSE means and standard deviations obtained from three datasets) are summarized in Table 3. From Table 3, we can see that overall, adding more LSTM units can achieve better results with obtained smaller RMSEs (by comparing the average RMSEs for $L_{N}=50$ and those for $L_{N}$ = 250). However, there is no obvious relationship between the network performance and the filter number ($F_{N}$). From the table, we can see that the optimal performance is obtained with an LSTM number 200 and filter number 250, with the smallest average mean RMSE being obtained (bolded in the table).

Moreover, we have also tested whether adding more LSTM layers or Residual blocks can further improve the performance, with the results being summarized in Table 4. From Table 4, we can see that adding more LSTM layers in LSTM–RNN or residual blocks in TCN deteriorates the performance for the majority of cases (only with a very marginal reduction in mean RMSE for dataset 2 by adding one LSTM layer). From this table, we can see that adding more LSTM layers or residual blocks does not gain extra benefits due to over-fitting.

**Figure 4:**
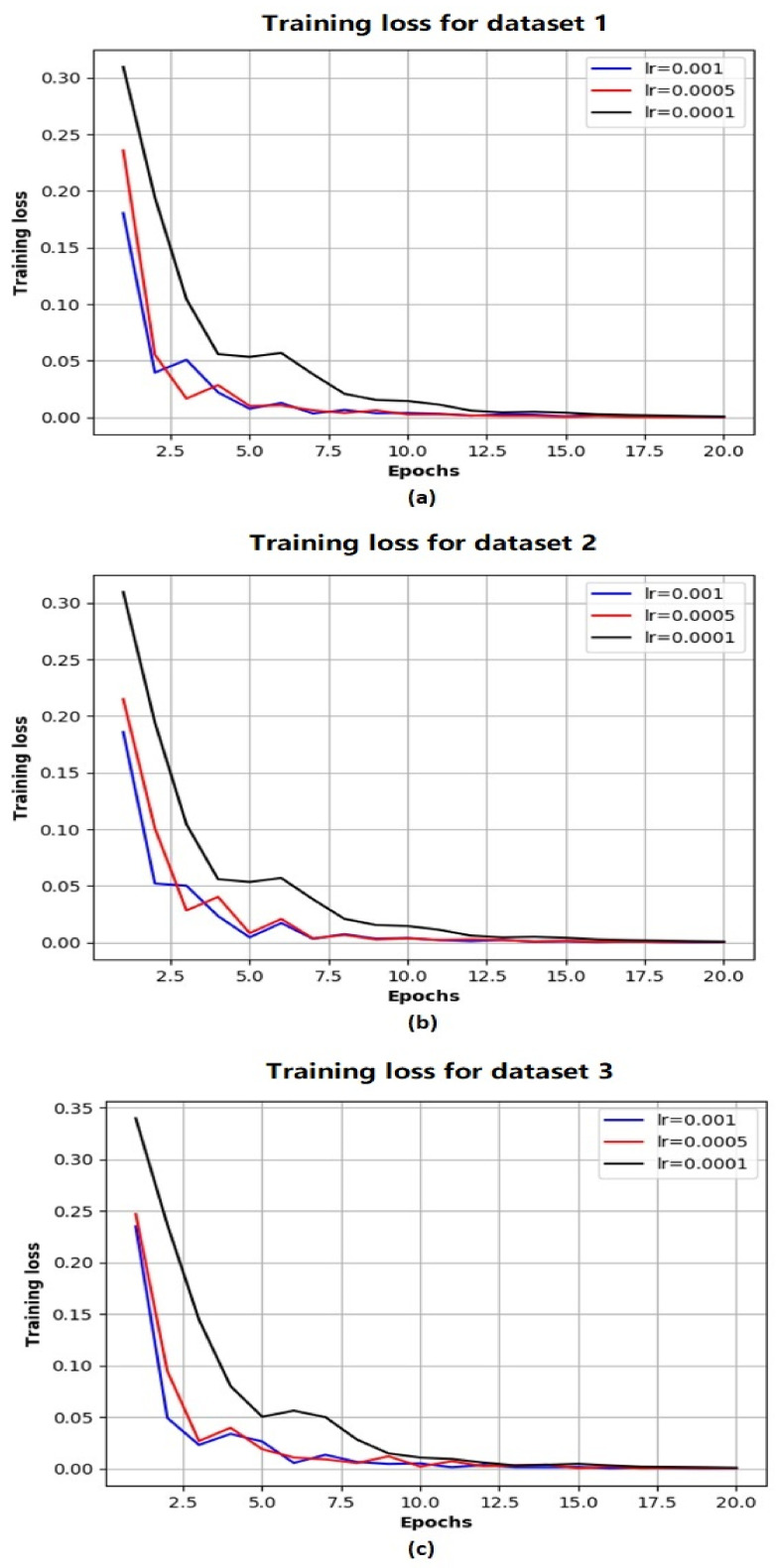
The evolution of MSE losses with training epoches with respect to Dataset 1 (**a**) Dataset 2 (**b**) Dataset 3 (**c**).

**Figure 5:**
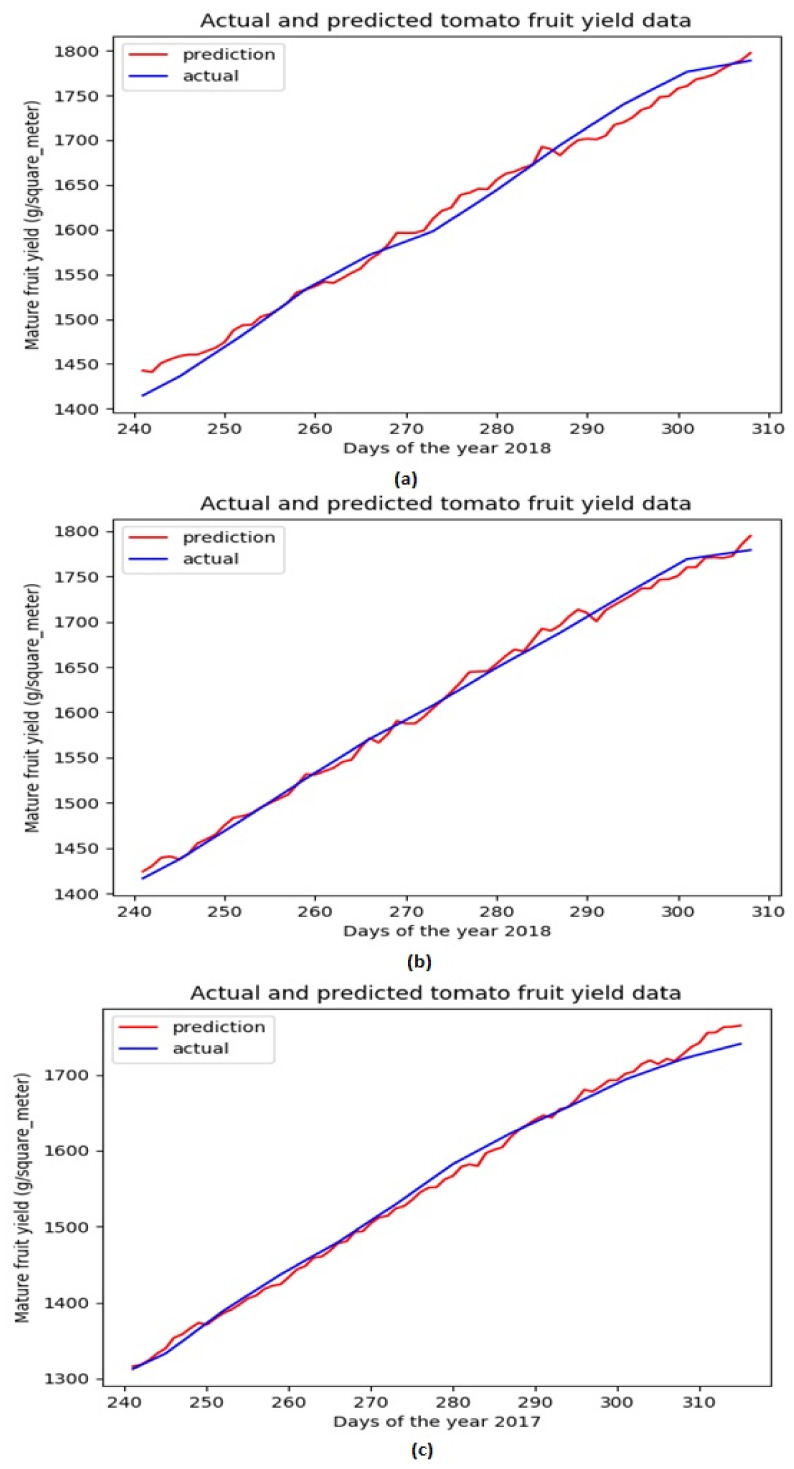
Ground truth tomato fruit yield values and predicted ones for testing datasets associated with Dataset 1 (**a**) Dataset 2 (**b**) Dataset 3 (**c**).

Based on the above evaluations, we have determined that the network architecture used in this study be with one LSTM layer and one TCN block. While the number of filters in the TCN block is chosen as 250 and that of the LSTM units is chosen as 200. Figure 4 shows the evolution of MSE losses with respect to training epoch for training our network model with the aforementioned architecture based on three training datasets. As the epoch increases, we can see that the MSE losses successfully converge to 0 for all the scenarios, while the convergence rate is fastest with the largest learning rate (when lr=0.001). Figure 5 shows the comparison of ground truth accumulated yields and predicted ones by our trained network during different time periods. We can intuitively observe that the predicted tomato fruit yield values almost coincide with the ground truth ones for all three scenarios.

Moreover, we have evaluated the importance of each factor for future yield prediction. In particular, at one time, we have excluded a factor as the network input and then trained/tested the network model on three datasets multiple times. The obtained averaged RMSE mean and standard deviation values are calculated and summarized in Table 5. We can obviously find out that the obtained error by excluding the historical yield information is much larger than those obtained by excluding other factors. Based on the results in Table 5, we can conclude that the historical yield information plays the most important role in the future yield prediction.

### 4.4. Comparison Studies

We have compared our developed DNN method with other methods, including both traditional machine learning-based methods (linear regression (LR), random forest (RF), support vector regression (SVR), decision tree (DT), gradient boosting regression (GBR), multi-layers artificial neural network (MLANN)) as well as other deep learning-based ones (single/multiple layer(s) LSTM–RNN [5], LSTM–RNN with attention mechanism [23] and TCN with single/multiple residual blocks). The comparison results are summarized in Table 6. From Table 6, we can see the majority of deep learning-based models (multiple layers LSTM–RNN, LSTM–RNN with attention, TCN with multiple blocks, and ours) outperform classical machine learning models with smaller mean RMSEs for all three datasets, which shows the advantages of the adopting of the deep learning for the greenhouse crop yield prediction. Furthermore, among the deep learning models, the proposed model in this work achieves the best performance with the smallest mean RMSEs for all three datasets.

## 5. Conclusions

In this work, we have proposed a new methodology for greenhouse crop yield prediction, by integrating two state-of-the-arts DNN network architectures used for temporal sequence processing: RNN and TCN. Given an input temporal sequence containing historical yield and environmental information, firstly, an LSTM—RNN is applied for pre-processing the original inputs to extract representative features, which are then further processed by a sequential of residual blocks of the TCN module. The features finally extracted from the TCN are then fed into a fully connected network for future crop yield prediction. Comprehensive evaluations through statistical analysis of obtained RMSEs for multiple datasets have shown that:

(i) The proposed approach can be applied for accurate greenhouse crop yield prediction, based on both historical environmental and yield information.
(ii) The proposed approach can achieve much more accurate prediction than other counterparts of both traditional machine learning and deep learning methods.

Furthermore, it is also shown in the experimental study that the historical yield information is the most important factor for accurately predicting future crop yields.

With respect to future work, to further validate the general effectiveness of the proposed model, we will evaluate it on more datasets collected from different growers on different sites. Moreover, we will also test the model’s performance on yield prediction for different types of popular greenhouse crops. More advanced network architecture will also be considered, for example, the LSTM encoder–decoder component as in [23] will be considered to be incorporated into the current model to build up a more advanced network architecture. Finally, we will also investigate the combination of the developed machine learning-based model with a biophysical model to achieve more accurate/robust crop yield prediction based on a multi-model framework.

## Figures and Tables

**Table 1:** Datasets descriptions.

	Dataset 1	Dataset 2	Dataset 3
Location	Greenhouse 1	Greenhouse 2	Greenhouse 2
Time period	2018	2017	2018
Information included	yield information (g/m${}^{2}$)		
	CO${}_{2}$ concentration (mmp)		
	temperature (${}^{\circ }$C)		
	humidity deficit (g/kg)		
	relative humidity (percentage)		
	radiation (W/m${}^{2}$)		

**Table 2:** Descriptive statistics of greenhouse environmental parameters associated with different datasets.

		Dataset 1	Dataset 2	Dataset 3
CO${}_{2}$ (mmp)	Min	535.97	370.94	478.05
	Max	1634.10	967.40	1691.43
	Median	793.95	629.97	769.79
	Mean	785.95	624.19	770.37
	Standard deviation	152.52	129.58	175.61
Temperature (${}^{\circ }$C)	Min	4.73	3.68	4.72
	Max	23.73	23.89	23.69
	Median	18.30	18.46	18.31
	Mean	17.25	17.01	17.18
	Standard deviation	3.97	4.25	3.94
Humidity deficit (g/kg)	Min	0.33	0.13	0
	Max	6.70	7.27	6.08
	Median	2.27	2.78	2.58
	Mean	2.91	2.91	2.65
	Standard deviation	1.40	1.29	1.33
Relative humidity (%)	Min	63.04	65.31	65.09
	Max	96.24	98.50	100
	Median	83.87	83.22	84.73
	Mean	82.49	82.19	83.99
	Standard deviation	6.57	5.88	6.72
Radiation (W/m${}^{2}$)	Min	0.59	0.58	0.59
	Max	82.91	83.02	82.91
	Median	42.81	43.41	42.81
	Mean	42.17	42.19	42.17
	Standard deviation	19.37	18.92	19.37

**Table 3:** Obtained mean and standard deviation of RMSEs with different LSTM unit numbers ($L_{N}$) and convolutional filter numbers ($F_{N}$) for three datasets.

		$L_{N}$	50	100	200	250
	$F_{N}$					
Dataset 1	50		16.20 ± 5.25	8.24 ± 0.78	13.84 ± 0.72	10.82 ± 0.80
	100		16.51 ± 0.87	11.45 ± 0.64	9.62 ± 0.04	9.96 ± 1.78
	200		19.57 ± 2.80	18.54 ± 1.57	16.30 ± 1.56	11.11 ± 0.13
	250		10.48 ± 0.64	16.99 ± 0.22	10.45 ± 0.94	9.98 ± 0.27
Dataset 2	50		8.91 ± 1.78	9.01 ± 0.73	7.16 ± 0.50	7.26 ± 1.21
	100		11.81 ± 1.22	8.47 ± 0.52	8.81 ± 1.85	8.23 ± 0.27
	200		10.62 ± 1.58	7.07 ± 1.90	7.96 ± 0.25	6.33 ± 1.48
	250		11.62 ± 0.02	8.78 ± 0.12	6.76 ± 0.45	7.95 ± 0.44
Dataset 3	50		11.96 ± 2.29	8.54 ± 0.71	8.21 ± 0.99	8.02 ± 0.20
	100		11.08 ± 4.61	8.58 ± 1.40	8.18 ± 0.44	8.88 ± 0.53
	200		8.85 ± 1.52	7.41 ± 1.48	8.67 ± 1.15	8.77 ± 1.20
	250		9.35 ± 1.57	7.46 ± 1.78	7.40 ± 1.88	10.06 ± 0.99
Average	50		12.36 ± 3.11	8.60 ± 0.74	9.74 ± 0.74	8.70 ± 0.74
	100		13.13 ± 2.27	9.50 ± 0.85	8.87 ± 0.78	9.02 ± 0.85
	200		13.01 ± 1.97	11.01 ± 1.65	10.98 ± 1.00	8.74 ± 0.94
	250		10.48 ± 0.74	11.08 ± 0.71	**8.20** ± 1.09	9.33 ± 0.57

**Table 4:** Mean and standard deviation of RMSEs for different LSTM layers and residual block numbers on different datasets.

		Layer Number	1	2
	Block Number			
Dataset 1	1		**10.45** ± 0.94	10.93 ± 2.73
	2		22.52 ± 10.08	15.58 ± 8.27
Dataset 2	1		6.76 ± 0.45	**6.50** ± 0.45
	2		9.18 ± 1.60	7.12 ± 0.18
Dataset 3	1		**7.40** ± 1.88	13.41 ± 2.09
	2		9.95 ± 0.72	16.85 ± 3.11

The smallest mean RMSE is marked bold.

**Table 5:** Statistical metrics (mean and standard deviation) of RMSEs (g/m${}^{2}$) by excluding certain input factors.

Excluding CO_2_ Concentration	11.88 ± 2.01
Excluding temperature	12.45 ± 0.87
Excluding HD	14.17 ± 2.98
Excluding RH	14.98 ± 3.88
Excluding radiation	12.84 ± 4.55
Excluding historical yield information	831.54 ± 73.02

**Table 6:** Statistical metrics (mean and standard deviation) of RMSEs (g/m${}^{2}$) obtained by different methodologies for three datasets.

		Dataset 1	Dataset 2	Dataset 3
Classical models	LR	23.77 ± 0	21.20 ± 0	17.88 ± 0
	RF	28.84 ± 1.02	27.69 ± 0.56	26.47 ± 1.44
	SVR	55.10 ± 0	46.62 ± 0	49.12 ± 0
	DT	28.93 ± 1.33	28.93 ± 1.96	27.03 ± 2.64
	GBR	28.93 ± 0.65	27.07 ± 0.52	23.98 ± 0.44
	MLANN	95.81 ± 43.33	60.27 ± 19.03	47.01 ± 13.37
DL models	LSTM–RNN (single layer) [5]	25.34 ± 5.62	13.12 ± 4.31	15.65 ± 4.01
	LSTM–RNN (multiple layers) [5]	14.16 ± 0.86	10.08 ± 0.84	12.38 ± 0.58
	LSTM–RNN with attention [23]	20.18 ± 1.87	13.20 ± 2.67	13.60 ± 1.50
	TCN	51.67 ± 29.87	30.79 ± 8.24	26.20 ± 7.54
	TCN (multiple blocks)	16.96 ± 0.76	14.12 ± 3.06	11.41 ± 5.61
	Ours	**10.45** ± 0.94	**6.76** ± 0.45	**7.40** ± 1.88

The smallest mean RMSE for each dataset is marked bold.

## Data Availability

Restrictions apply to the availability of these data. Data was obtained from a third party collaborator of the SMARTGREEN project and are available under permissions.
